# The Effects of Pedaling-Based Progressive Resistance Training on Range of Motion, Muscle Strength, and Physical Function in Female Patients with Total Knee Arthroplasty: Single-Blind Randomized Controlled Trial

**DOI:** 10.3390/medicina61081441

**Published:** 2025-08-10

**Authors:** Jungae An, Junseok Seo, Byoung-Hee Lee

**Affiliations:** 1 Graduate School of Physical Therapy, Sahmyook University, Seoul 01795, Republic of Korea; ajapt@syu.ac.kr (J.A.); smse87@naver.com (J.S.); 2 Department of Physical Therapy, Sahmyook University, Seoul 01795, Republic of Korea

**Keywords:** total knee arthroplasty, muscle strength, range of motion, function

## Abstract

*Background and Objectives*: Total knee arthroplasty (TKA) is an effective solution for pain relief and functional recovery in patients with end-stage osteoarthritis. However, stiffness of the knee, limited range of motion, and weakened muscle strength are challenges of postoperative rehabilitation. This study investigated the effects of a pedaling-based progressive resistance training (PPRT) program on range of motion, muscle strength, physical function, and gait in patients who had undergone TKA. *Materials and Methods*: A total of 48 female patients (aged 65–79) who underwent TKA participated in the study and were randomly assigned to either the PPRT group (*n* = 24) or the control group (*n* = 24). The PPRT group performed the training for 60 min per session, twice a day, five days a week, for four weeks. The primary outcomes were the muscle strength and range of motion (ROM) of the knee. Secondary outcomes included the Western Ontario and McMaster Universities Osteoarthritis Index (WOMAC) and the Timed Up and Go (TUG) test. *Results*: There was a significant time × group interaction effect in all the quadriceps strength values (*p* < 0.05), with a moderate to large effect size (*η*^2^*_p_* = 0.142–0.390). The PPRT group showed a smaller decrease in knee flexor and extensor strength and a greater improvement in knee flexion range of motion compared with the control group (*p* < 0.05). The WOMAC index and TUG time were also significantly improved compared with the control group (*p* < 0.05). In the time × group interaction, a significant effect was shown in WOMAC pain, physical function, and total score (*p* < 0.05) with a moderate to large effect size (*η*^2^*_p_* = 0.099–0.196). TUG time also showed a significant time × group interaction (*p* < 0.05) with a moderating effect (*η*^2^*_p_* = 0.0840). *Conclusions*: This study suggests that pedaling-based progressive resistance training helps maintain knee flexor and extensor strength as well as improves range of motion and physical function in patients following TKA and can be proposed as effective training for post-TKA rehabilitation.

## 1. Introduction

Total knee arthroplasty (TKA) is a commonly used surgical intervention when knee arthritis fails to improve with physical therapy and conservative treatments, aiming to reduce pain and restore physical function [1]. TKA accounts for about 51% of all total joint arthroplasty procedures, and recent advancements in surgical techniques and implant technologies could profoundly enhance functional activity levels for TKA patients [2]. However, 10% of patients following TKA may still be dissatisfied due to experiencing symptoms such as reduced muscle strength, atrophy of the knee extensor and flexor muscles, and joint stiffness for up to one year after TKA [3,4,5]. The patients following TKA tend to remain sedentary due to pain or physical dysfunction, avoid prolonged activity, and walk significantly less than people of the same age [6,7]. This decline in knee extensor and flexor muscle function reduces walking ability and overall knee function as well as increases the risk of accidents and, in severe cases, even death due to falls [8,9]. Therefore, it is essential to engage in training that improves muscle strengthening, physical function, aerobic activity, and joint range of motion [10].

Currently, patients who have undergone TKA are managed with various interventions, including range of motion exercises, resistance training, weight-bearing exercises, balance training, and preoperative exercise programs aimed at improving physical function [11]. These interventions are designed to enhance gait, muscle strength recovery, balance, and overall functional movement [12]. However, a stiff knee gait pattern observed after TKA is caused by limited knee joint range of motion, pain, and reduced strength in the knee extensor and flexor muscles. This abnormal gait pattern can result in excessive mechanical loading on the prosthetic joint, compromise knee stability, and increase the risk of revision surgery. Therefore, an exercise program specifically aimed at strengthening the knee extensors and flexors is essential [13,14,15]. Furthermore, patients with unilateral TKA may develop lower extremity imbalances along with muscle weakness, potentially accelerating osteoarthritis in the unaffected knee [16,17].

Progressive resistance training has been employed as a highly effective method for improving muscle strength and bone mineral density in the elderly [18]. Progressive resistance training is a method of starting with a low intensity and gradually increasing the intensity, which can be an effective approach to restore muscle strength and function following TKA [13]. Pedaling exercise, in particular, offers several advantages: it helps increase the range of motion of the knee, strengthens the quadriceps femoris while applying less than half the force to the tibia compared with walking, imposes less load on the knee joint than weight-bearing exercises, reducing stiffness, and activates lower extremity muscles [19,20,21]. It is widely used to improve physical function in patients with knee arthritis who must avoid excessive weight-bearing, and when performed in water, pedaling exercise has been shown to reduce pain, improve physical function, and enhance muscle strength [22]. Furthermore, pedaling exercise has been demonstrated to effectively alleviate pain and swelling in patients with TKA [21,23].

Therefore, combining pedaling exercise with progressive resistance training may enhance physical function, restore muscle strength, and improve gait ability in patients following TKA [18,21,23]. The purpose of this study was to evaluate the effects of pedaling-based progressive resistance training on range of motion, muscle strength, physical function, and gait in patients who underwent total knee arthroplasty, and to propose an effective rehabilitation method based on the measured outcomes.

We hypothesized that pedal-based progressive resistance training would be effective in improving range of motion, knee flexion and extension strength, and physical function in patients following TKA. Therefore, this single-blind, randomized controlled trial was conducted to evaluate the effects of pedal-based progressive resistance training on postoperative recovery in these patients.

## 2. Materials and Methods

### 2.1. Participants

The participants of this study were 48 female patients who underwent total knee arthroplasty at an orthopedic surgery and rehabilitation hospital.

The inclusion criteria for study participants were as follows: female patients aged 65 years or older; individuals capable of clear verbal communication; patients diagnosed with end-stage osteoarthritis based on radiographic or functional findings; and those who had undergone cemented total knee arthroplasty. The exclusion criteria for this study were as follows: patients aged 80 years or older; those with a history of knee surgery prior to total knee arthroplasty; patients with physical impairments that made walker use difficult; individuals with neurological disorders or a mental illness; and those with a history of spinal surgery [24].

The required sample size was calculated using G*Power Version 3.1.9.7 (Franz Faul, University of Kiel, Kiel, Germany, 2020). Based on a repeated measures analysis of variance (ANOVA), which was the primary statistical method used to evaluate the effectiveness of the program, a total of 44 participants were required to achieve an effect size of 0.25, a significance level (α) of 0.05, and a statistical power of 0.95 [25]. Considering a dropout rate of 15%, 50 participants were recruited, and all participants were randomly assigned to two groups using the Research Randomizer program (http://www.randomizer.org/, accessed on 26 September 2022) after completing a pre-test. During the study, one person from each group dropped out, and a total of 48 participants’ data were used for analysis.

### 2.2. Ethical Statement

All participants received a clear and comprehensible written explanation of the study’s purpose and procedures before their participation. They were also informed that they could withdraw their consent to participate at any time during the study. Written informed consent was obtained from all participants before the study commenced. This study was approved by the Research Ethics Review Committee of Sahmyook University (Approval Number: SYU 2022-07-017-002, Approval Date: 31 August 2022) and is registered with the Clinical Research Information Service (CRIS) under registration number KCT0007884. Participants’ rights were protected according to the ethical principles of the Declaration of Helsinki.

### 2.3. Study Procedure

#### 2.3.1. Surgical Procedure

This single-blind randomized controlled trial was conducted in the inpatient acute care unit of an orthopedic surgery hospital in South Korea from September 2022 to June 2023 (including from IRB approval to post-test of final participants). All participants underwent robot-assisted TKA performed by a senior surgeon using a tourniquet, and a minimally invasive midline skin incision, followed by a mid-vastus approach. A high-flexion mobile prosthesis (Implantcast; GmbH, Lüneburger Schanze, Buxtehude, Germany) was used, and all implants were fixed with cement. Postoperative rehabilitation, such as a cold pack, continuous passive motion (CPM), intermittent pneumatic compression (IPC) therapy, and an ankle pump, was started on the day of TKA. After the removal of the drain on the second postoperative day, the patients wore compression stockings and a knee brace and began bearing their weight with a walker.

#### 2.3.2. Experimental Procedure

This study was conducted by five physical therapists who had more than 5 years of musculoskeletal field experience. All research members underwent a one-hour training session one week prior to the start of the intervention. The training covered a general overview of the experimental procedures, measurement protocols, and proper use of the measurement equipment. The experiment was conducted in a fully informed and standardized manner to ensure consistency and accuracy. The assessors and participants were blinded to group assignments. Both baseline and post-intervention tests were conducted by the same examiner, who was trained to use the measuring equipment sufficiently before assessing patients.

The experimental group, which performed pedaling-based progressive resistance training, completed a total of 40 sessions, each lasting 60 min, conducted twice daily, five days per week, for four weeks, beginning on the third day after TKA. The control group performed only progressive resistance training and completed a total of 40 sessions, each lasting 30 min, conducted twice daily, five days per week, for four weeks, beginning on the third day after TKA. In addition, both groups received general physical therapy, including active and passive ROM exercise and continuous passive motion (CPM) therapy once daily for 20 min, five days per week, for four weeks.

The baseline assessment was performed 2 weeks before the TKA, and the post-intervention test was performed 6 weeks after the TKA. The range of motion was measured using an electronic goniometer. Muscle strength before and after the intervention was evaluated using the Biodex System 3 (Biodex 3Pro830-210, Biodex Medical Systems Inc., Shirley, NY, USA). Physical function was assessed using the Western Ontario and McMaster Universities Osteoarthritis Index (WOMAC). Gait ability was measured through a Timed Up and Go (TUG) test using a chair with armrests. The experimental intervention and assessments were conducted accordingly.

### 2.4. Intervention

#### Pedaling-Based Progressive Resistance Training

**Figure 1:**
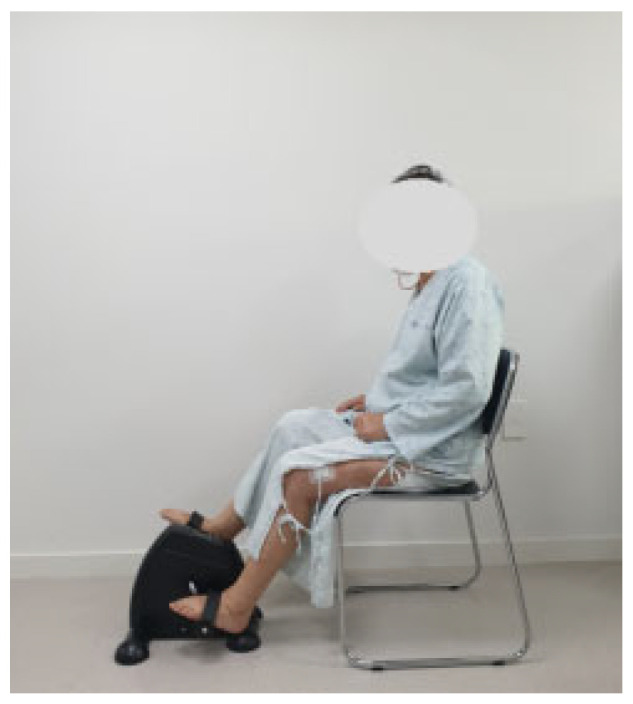
Pedaling exercise.

**Figure 2:**
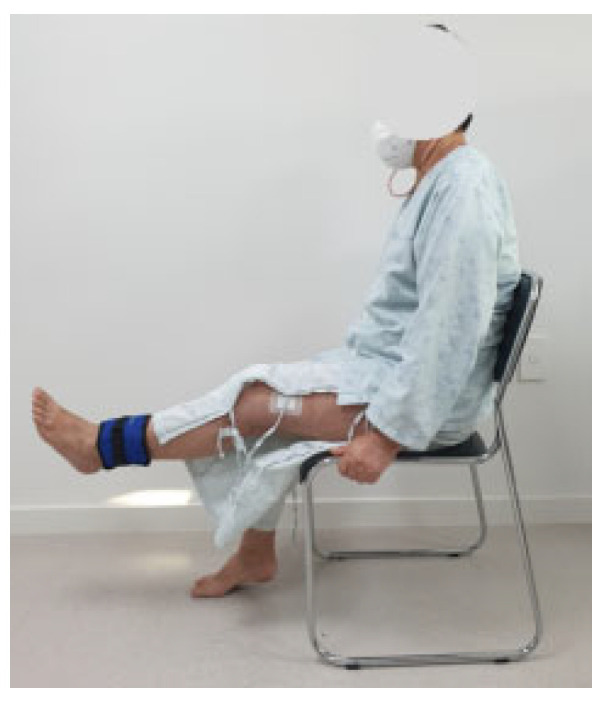
Progressive resistance exercise with sandbag.

The pedal-based progressive resistance training program was designed based on previous studies and was gradually progressed week by week, taking into account patient safety and exercise difficulty [21,26,27,28]. A stationary floor pedaling device was used for the pedaling exercise (Figure 1), and resistance was provided using sandbags weighing between 500 g and 5 kg (Figure 2). The pedaling exercise protocol consisted of a 5 min warm-up, 30 min of main exercise, and a 5 min cool-down. The pedal-based progressive resistance exercise began three days after TKA, and the detailed program is outlined in Table 1. During weeks 1 and 2, training included ankle exercises, straight leg raises, quadriceps sets, quadricep arcs, thigh squeezes, and knee joint range-of-motion exercises, performed in 3 sets of 10 repetitions each, alongside pedaling exercises at a basic intensity for 30 min. In week 3, the same set of exercises was performed using sandbags at 50% of one-repetition maximum (1RM) for 3 sets of 10 repetitions. Pedaling exercise intensity was increased to 40–60% of heart rate reserve following the warm-up. During week 4, the exercises were performed at 70% of 1RM for 3 sets of 10 repetitions, with pedaling exercises maintained at 40–60% of heart rate reserve after warm-up. A rest period of 30–40 s was allowed between sets (Table 1).

### 2.5. Outcome Measurements

#### 2.5.1. Primary Outcomes

The primary outcomes in this study are ROM and muscle strength.

The knee flexion range of motion was measured using an electronic goniometer (Biometrics, Baton Rouge, LA, USA, 2008) with excellent reliability (knee flexion ICC = 0.994, knee extension ICC = 0.978–0.987) [29]. To minimize measurement errors, the goniometer was calibrated to 0 degrees prior to measurement. The active range of motion was measured three times consecutively, and the average value was calculated. The examiner sat on a chair level with the bed where the measurement was performed. The axis of the goniometer was aligned with the lateral articular surface of the tibia, the fixed arm was positioned along the midline of the femur from the greater trochanter, and the moving arm was aligned from the head of the fibula toward the lateral malleolus. The subject was instructed to actively flex their knee, and the measurement was taken within the pain-free range of motion, with the subject asked to stop if pain was experienced [30]. A minimal detectable change (MDC) was reported as 9.6° of knee flexion ROM in TKA patients [31]. Muscle strength was measured using the Biodex System 3 (Biodex 3Pro830-210, Biodex Medical Systems, Shirley, NY, USA), assessing the maximum strength of the knee extensors and knee flexors. The joint measurement posture followed the protocol outlined in the Biodex manual. Biodex System 3 is a highly reliable muscle strength measurement device, with an ICC of 0.95 [32]. To measure the strength of the knee extensors and flexors, the rotation axis of the dynamometer was aligned with the subject’s knee joint axis, and the chest, abdomen, and thighs were secured using adjustment belts. A strap was fastened 1 cm above the lateral malleolus, which served as the point of force application during knee extension. The subject then performed knee extension movements. Measurements were conducted by a therapist with five years of experience, who provided auditory cues and visual feedback via a monitor. Five maximal contractions were performed at angular velocities of 60°/s and 180°/s, with the range of motion set from 10° to 90° of knee flexion. A total of five measurements were taken, with a 2 min rest period between trials. The average value of these five measurements was used for analysis [33]. There are no reports about the minimum clinically important difference (MCID) for muscle strength after TKA.

#### 2.5.2. Secondary Outcomes

The Western Ontario and McMaster Universities Osteoarthritis Index (WOMAC) was used to assess self-reported physical function. The WOMAC, widely used globally for patients with arthritis, consists of 27 items divided into three subscales addressing physical function problems encountered in daily life, and demonstrates high test–retest reliability for TKA (pain ICC = 0.79–0.92, stiffness ICC = 0.76–0.88, and physical function ICC = 0.71–0.95) [34]. The advantage of the WOMAC is its efficiency in identifying functional disability and pain in patients with knee arthritis due to its concise questions and detailed scaling compared with other assessment tools [35]. In the WOMAC, the pain subscale evaluates pain during activities such as walking, climbing stairs, and lying in bed, with scores ranging from 0 to a maximum of 20 points. The joint stiffness subscale is scored from 0 to a maximum of 8 points, while the physical function subscale, which includes activities such as using stairs and standing up from a sitting position, is scored from 0 to a maximum of 68 points. When combined, the total possible score across all three subscales ranges from 0 to 96 points. For each item, 0 indicates no pain or difficulty, and 4 means very severe pain or impairment. A higher total score corresponds to greater levels of pain, stiffness, and physical disability in daily life. The MCID for the WOMAC score in TKA patients has been suggested, with subscale values ranging from 13.3 to 36.0 for pain and 1.8 to 33.0 for function [36]. The Timed Up and Go (TUG) test was used to assess walking ability. The TUG test is commonly employed to evaluate mobility in elderly patients [37] and serves as a tool to measure walking ability, balance, and fall risk. The TUG test has demonstrated a high test–retest reliability, with an intraclass correlation coefficient (ICC) ranging from 0.98 for TKA [38]. During the test, two chairs with armrests are placed 3 m apart, and the subject sits on one of the chairs. The subject is then instructed to stand up, walk to the opposite chair, turn around, and sit back down on the original chair. This procedure is repeated three times, and the average time is calculated [39]. The MCID for the TUG time in TKA patients was reported as 2.27 s [38].

### 2.6. Statistical Analysis

All data analyses in this study were performed using SPSS version 27.0. Means and standard deviations were calculated for all variables. The normality of the variables was assessed using the Kolmogorov–Smirnov test, which confirmed that all variables followed a normal distribution. Repeated measures ANOVA was conducted to examine interaction effects between time (pre- vs. post-intervention) and group (experimental vs. control). The effect size (*η^2^_p_*: 0.01 small, 0.06 medium, 0.14 large) was calculated from the repeated measures ANOVA to assess both statistical significance and clinical relevance. Additionally, based on the results of the independent *t*-test for pre- to post-intervention changes, the effect size (Cohen’s *d*: 0.2 small, 0.5 medium, 0.8 large) and its 95% confidence interval (CI) were calculated [40]. Statistical significance was set at a *p*-value of 0.05 for all analyses.

## 3. Results

Statistical analysis was conducted on the experimental results of 48 female participants (24 in the PPRT group and 24 in the control group) who underwent TKA. The general characteristics and homogeneity test of the study participants are presented below. The results confirmed that both the pedaling-based progressive resistance exercise group and the control group were homogeneous prior to the intervention (Table 2).

### 3.1. Primary Outcomes

In the time × group interaction, no significant effect was shown at knee ROM, *F*(1,46) = 4.016, *p* = 0.051. The effect size, Cohen’s *d*, was 0.579, and *η*^2^***_p_*** was 0.080; both values indicate a moderating effect. The PPRT group showed a significant improvement from 115.58° before training to 131.97° after training (*p* < 0.001), and the control group showed a significant improvement from 120.83° before training to 129.51° after training (*p* < 0.01). However, in the comparison of differences between groups, the PPRT group showed no significant difference compared with the control group (*p* = 0.051) (Table 3).

The changes in the strength of the knee extensors and knee flexors in the PPRT group and the control group after 4 weeks of training are shown in Table 4 and Table 5. There was a significant interaction effect between time and group in all the quadricep strength values (*p* < 0.05), with a moderate to large effect size (*η*^2^***_p_*** = 0.142–0.390). In addition, in the muscle strength variables of the knee extensors, there was a significant difference between the groups (*p* < 0.05, Cohen’s *d =* 0.743–0.815) at FLX60°/s PT (N-M) and FLX60°/s PT/BW (%) and the time × group interaction effect (*p* < 0.05), with a moderate to large effect size (*η*^2^***_p_*** = 0.126–0.164) at FLX60°/s PT (N-M) and FLX60°/s PT/BW (%). The two groups showed significant differences in all variables of the knee extensor muscles (*p* < 0.05). The PPRT group maintained muscle strength or showed a mild decrease in muscle strength, whereas the control group showed a rapid decrease in muscle strength. In the muscle strength variables of the knee flexors, there was a significant difference between the groups (*p* < 0.05, Cohen’s *d =* 0.797–1.567).

### 3.2. Secondary Outcome

In the time × group interaction, a significant effect was shown at WOMAC pain, physical function, and total score (*p* < 0.05) with a moderate to large effect size (*η*^2^*_p_* = 0.099–0.196).

Both groups showed significant differences after the intervention in the WOMAC subscales of pain, stiffness, and physical function (*p* < 0.05). In the between-group comparison, there was a significant difference in pain and physical function (*p* < 0.05); however, there was no significant difference in stiffness (*p* = 0.150). The effect size (Cohen’s *d*) ranged from 0.423 to 0.967.

In the TUG time of the time × group interaction, a significant effect was shown, *F*(1,46) = 4.216, *p* = 0.046. The effect size, Cohen’s *d*, was 0.596 and *η*^2^*_p_* was 0.084, indicating a moderating effect. The PPRT group showed a significant improvement from 12.96 s before training to 9.32 s after training (*p* < 0.001), and the control group showed a significant improvement from 12.75 s before training to 10.31 s after training (*p* < 0.001). In addition, when comparing the differences between groups, the PPRT group showed a significant difference compared with the control group (*p* < 0.05) (Table 6).

## 4. Discussion

This study investigated the effects of a 4-week pedaling-based progressive resistance training on the range of motion, muscle strength, and physical function in female patients with TKA. After the 4-week intervention, the PPRT group showed greater improvements across all measured variables compared with the standard progressive resistance exercise group.

### 4.1. Primary Outcomes

Patients who underwent TKA experienced a 10% reduction in the cross-sectional area of the quadriceps femoris, a 17% decrease in voluntary muscle activity, and up to an 85% loss of muscle mass postoperatively compared with preoperative levels [41]. Early initiation of strength training after TKA can reduce knee pain, restore range of motion, improve lower extremity strength, and prevent knee stiffness [42].

In this study, the knee ROM in the PPRT group increased from 115.58° to 131.97° after intervention, showing a significant improvement of 16.39° (*p* < 0.001). The control group also showed a significant improvement of 8.67° (*p* < 0.001). However, there was no significant difference between the PPRT and control groups. The MDC reported for TKA patients is 9.6° of knee flexion ROM, and the PPRT group exceeded this threshold with a post-intervention increase of 16.39° (14.19%) [31]. Sattler et al. [21] conducted a pedal-based exercise protocol on 60 patients with early-stage TKA and found no significant difference in knee ROM compared with the non-pedaling protocol group. This is revealed to be because pedaling does not utilize the full range of motion of the knee joint during exercise for patients with TKA [43], resulting in no significant difference compared with the control group. Pozzi et al. [44] performed progressive strengthening training on 165 patients undergoing TKA and confirmed significant improvements in knee joint range of motion compared with the control group (*p* < 0.001). Compared with the standard group, the progressive strengthening training showed better results in knee extension ROM and quadricep strength (*p* < 0.05). This is likely because ankle exercises, leg-lifting exercises, and leg-pressing exercises during progressive resistance training help prevent muscle contraction delay and activate the knee extensors and flexors, leading to a significant difference in joint range of motion (*p* < 0.001).

Wei-Hsiu et al. [25] applied a hospital-based progressive resistance exercise for 24 weeks in female patients after TKA and found that it significantly improved lower extremity muscle strength compared with the control group, specifically in knee extensor and knee flexor muscle strength (*p* < 0.05). Sanzo et al. [23] applied an active-assisted cycle ergometer for 6–12 weeks in TKA and investigated the effect compared with the standard care group or control group. The active-assisted cycle ergometer group showed no significant difference compared with the standard care group or the control group in resisted isometric knee flexor and extensor strength, but the knee extension force recovered to a level similar to that before TKA.

In the results of this study for the knee extensors, no increase in muscle strength was observed in the pre- and post-intervention comparison within the PPRT group. However, in comparing the PPRT group with the control group, the PPRT group achieved preoperative muscle strength levels in EX60°/s PT (Nm), increasing from 38.49 to 39.10, while the control group decreased from 42.18 to 20.88. In EX60°/s PT/BW (%), the PPRT group saw a slight increase from 59.17 to 60.39, whereas the control group dropped from 73.92 to 35.31. In EX180°/s PT (Nm), the PPRT group also increased marginally from 59.17 to 60.39, while the control group fell from 73.92 to 35.31. In EX180°/s PT/BW (%), the PPRT group declined from 43.63 to 32.50, and the control group decreased from 50.85 to 23.50, revealing significant differences across all comparisons. For the knee flexor results, no increase in muscle strength was noted before and after the experiment. In FLX60°/s PT (Nm), the PPRT group decreased from 25.33 to 25.32, and the control group declined from 26.46 to 17.79. In FLX60°/s PT/BW (%), the PPRT group fell from 39.19 to 35.89, and the control group decreased from 46.16 to 29.93, demonstrating significant differences in both. However, in FLX180°/s PT (Nm), the PPRT group dropped from 19.71 to 13.64, and the control group decreased from 18.09 to 11.44. In FLX180°/s PT/BW (%), the PPRT group decreased from 30.42 to 14.00, and the control group fell from 32.01 to 18.75, showing no significant difference between the groups. Although there is no reported evidence on the MCID for quadricep strength in TKA patients, it is known that most patients experience a decline in strength following TKA. The results of this study suggest that PPRT may be effective in maintaining or improving quadricep strength postoperatively. Berghmans et al. [28] applied a progressive strength exercise for 6 weeks, leading to significant improvements compared with the control group, except for the knee flexor at 180° angular velocity in the female patient group (*p* < 0.05). The enhancement in lower extremity muscle strength appears to be due to the activation of the knee extensors through the pedaling exercise and progressive resistance training, alongside the leg extension and strain exercises, leg lifting exercises, and knee extension exercises. Pedaling exercise stimulated the motor response of the knee extensors and knee flexor groups, resulting in improved muscle strength. The absence of a significant difference in knee flexor strength at an angular velocity of 180° between the PPRT group and the control group indicates challenges in rapidly recovering knee flexor strength at this angular velocity during the early stages of TKA.

### 4.2. Secondary Outcomes

To perform functional movements in daily life after TKA, appropriate intensity exercises at the right time [45] and exercises with a reduced load on the knee joint are required [46]. Among the exercise programs that effectively improve physical function, including the above factors, PPRT should be considered.

In a previous study about an active-assisted cycle ergometer (AACE) for 6–12 weeks for patients undergoing TKA, the AACE group and the standard care control group showed significant improvements in knee function scores [23]. Although there was no significant difference between groups, the AACE group demonstrated higher knee function scores. This suggests that AACE, which provides active-assisted cycling exercise, may help improve knee function while minimizing pain and swelling. Rafiq et al. [47] applied progressive resistance strength training to patients with knee osteoarthritis for 12 weeks and showed significant differences compared with the control group in the WOMAC scores for quality of life and walking speed tests for functional capacity (*p* < 0.05). These results suggested that progressive resistance strength training appropriately applied to patients who underwent TKA was a way to improve physical function in daily life. Sattler et al. [21] applied a 16-week pedaling-based exercise protocol for acute-phase TKA patients, and the results were compared with the non-pedaling protocol group. The study found that the pedaling-based exercise protocol was superior to the non-pedaling protocol in functional and self-reported outcomes (*p* < 0.05).

In this study, the self-reported WOMAC questionnaire was used to assess subjective physical function in patients who underwent TKA, including items on pain, stiffness, and physical function. The pain score in the PPRT group decreased by 7.96 (59.3%) from 13.41 to 5.45, the stiffness score decreased by 2.37 (43.8%) from 5.41 to 3.04, and the physical function score decreased by 19.21 (38%) from 49.62 to 30.41 after the intervention. There was a significant improvement in function in both the PPRT group and the control group (*p* < 0.001). In the comparison between groups, the PPRT group showed significantly greater improvements compared with the control group (*p* < 0.01). The total WOMAC score in the PPRT group decreased by 29.54 (43.1%) from 68.46 to 29.54. The MCID for the WOMAC total score has not been reported, but the minimal clinical important improvement (MCII) in patients with osteoarthritis has been reported to be 12% [48]. As shown in previous studies, a significant difference in WOMAC scores was found in this study. This is because the pedaling exercise contributed to pain reduction, and the leg-raising and knee-extension exercises provided appropriate resistance to the rapid loss of muscle strength and function within one month, which is characteristic of patients who underwent TKA [11]. Pedaling exercise activated the knee extensors and knee flexors [44], which helped patients perform functional activities in daily life during the early postoperative period [49]. In the long term, early pedaling exercises and progressive resistance training will help restore lower extremities strength and facilitate social reintegration. We investigated changes in gait variables by measuring the time taken by patients to perform the TUG test. There was a significant difference in the TUG test between the PPRT group and the control group (*p* < 0.001). The PPRT group significantly decreased by 3.64 s (28%) from 12.96 s to 9.32 s (*p* < 0.001), and the PPRT group showed a significant improvement in gait ability compared with the control group (*p* < 0.05). The MCID for the TUG time in TKA patients was reported as 2.27 s, and the PPRT group achieved this threshold point [38]. This suggests that the pedaling exercise in this study improved the strength of the quadriceps femoris, gluteus maximus, and calf muscles in the TKA side [46] and reduced pain during walking [21], thereby enhancing the propulsive power of walking and improving walking speed.

The present study had several limitations. All study participants were females aged 65–80 patients from a specific country, so it is difficult to generalize the study results. This study consisted of a short-term 4-week program, which may be too short a period to evaluate long-term functional recovery, and long-term training is needed to stably induce structural changes in early neuromotor function after TKA surgery. There is also a lack of information on the long-term sustainability of training effects. Therefore, long-term follow-up observations are needed to assess the sustainability of training effects. In a future study, considering these limitations, a long-term training program should be applied to all patients who underwent TKA to standardize the training effect, and a long-term follow-up design is needed to verify the long-term effect.

## 5. Conclusions

Through this study, we confirmed that pedaling-based progressive resistance exercise is effective for knee ROM, muscle strength, and physical function in rehabilitation after TKA. The results of this study can be used to develop various rehabilitation programs for TKA patients and to expand the potential for clinical application.

## Figures and Tables

**Table 1:** Pedaling-based progressive resistance training program.

Week	Contents of the Program	Intensity
1st week	1. Ankle Exercise 2. Straight Leg Raises 3. Quadricep Sets 4. Quadricep Arcs 5. Thigh Squeezes 6. Knee ROM Exercise 7. Pedal Exercise	10 times, 3 sets Pedal Exercise 30 min
2nd–3rd week	1. Ankle Exercise 2. Straight Leg Raises 3. Quadricep Sets 4. Quadricep Arcs 5. Thigh Squeezes 6. Knee ROM Exercise 7. Pedal Exercise	10 times, 3 sets (50% of 1RM) Pedal Exercise 30 min 30–40% of heart rate reserve
4th week	1. Ankle Exercise 2. Straight Leg Raises 3. Quadricep Sets 4. Quadricep Arcs 5. Thigh Squeezes 6. Knee ROM Exercise 7. Pedal Exercise	10 times, 3 sets (70% of 1RM) Pedal Exercise 30 min 70% of heart rate reserve
Total training time is 60 min, and rest time between sets is 30–40 s.		

**Table 2:** General Characteristics and homogeneity test of study participants (*N* = 48).

Characteristics	PPRT Group (*n* = 24)	Control Group (*n* = 24)	*t*(*p*)	*X*^2^(*p*)
Age (years)	71.25 ± 6.22	71.83 ± 4.44	−0.374 (0.711)	
Height (cm)	154.84 ± 7.39	152.82 ± 5.60	1.067 (0.292)	
Weight (kg)	63.90 ± 7.18	62.73 ± 7.26	0.563 (0.576)	
BMI	26.63 ± 2.27	26.78 ± 2.18	−2.26 (0.822)	
Kellgren–Lawrence grade (III: IV)	9/15	8/16		0.091 (0.763)
Affected Side (Left: Right)	17/7	13/11		1.422 (0.233)

PPRT, pedaling-based progressive resistance training and BMI, body mass index. Values are expressed as mean ± standard deviation. Significant: *p* < 0.05.

**Table 3:** The comparison of knee ROM (*N* = 48).

Variable		PPRT Group (*n* = 24)	Control Group (*n* = 24)	Cohen’s *d* (95% CI)	Time	Group	Time × Group
					*F*(*p*)	*F*(*p*)	*F*(*p*)
ROM (°)	Pretest	115.58 ± 13.95	120.83 ± 12.62	0.579 (−0.002~1.153)	42.440 (0.000)	0.345 (0.560)	4.016 (0.051)
	Posttest	131.97 ± 5.35	129.51 ± 8.07				
	Mean difference	16.39 ± 11.73	8.67 ± 14.75				
	*t*(*p*)	−6.844 (0.000)	−2.881 (0.008)				
	Effect size (*η*^2^*_p_*)				0.480	0.007	0.080

Data are mean (standard deviation). PPRT, pedaling-based progressive resistance training and ROM, range of motion. The effect size (Cohen’s *d*) was calculated by comparing the pre- and posttest ROM means of both groups. *F*, repeated measures ANOVA. Significant: *p* < 0.05.

**Table 4:** Comparison of the quadricep muscle strength (*N* = 48).

Variable		PPRT Group (*n* = 24)	Control Group (*n* = 24)	Cohen’s *d* (95% CI)	Time	Group	Time × Group
					*F*(*p*)	*F*(*p*)	*F*(*p*)
EX60°/s PT(N-M)	Pretest	38.49 ± 16.04	42.18 ± 17.69	1.447 (0.803~2.079)	21.989 (0.000)	4.186 (0.047)	24.651 (0.000)
	Posttest	39.10 ± 13.87	20.88 ± 8.65				
	Mean difference	0.81 ± 9.53	−21.30 ± 19.39				
	*t*(*p*)	−0.312 (0.758)	5.381 (0.000)				
	Effect size (*η*^2^*_p_*)				0.323	0.083	0.349
EX60°/s PT/BW (%)	Pretest	59.17 ± 20.37	73.92 ± 32.63	1.567 (0.911~2.210)	25.944 (0.000)	0.944 (0.336)	29.456 (0.000)
	Posttest	60.39 ± 17.62	35.31 ± 14.45				
	Mean difference	1.22 ± 14.72	−38.61 ± 32.80				
	*t*(*p*)	−0.4.08 (0.687)	5.766 (0.000)				
	Effect size (*η*^2^*_p_*)				0.361	0.020	0.390
EX180°/s PT(N-M)	Pretest	28.35 ± 9.97	28.97 ± 11.27	0.797 (0.205~1.382)	63.544 (0.000)	2.271 (0.139)	7.628 (0.008)
	Posttest	21.03 ± 6.93	13.90 ± 6.66				
	Mean difference	−7.31 ± 7.53	−15.07 ± 11.51				
	*t*(*p*)	4.757 (0.000)	6.414 (0.000)				
	Effect size (*η*^2^*_p_*)				0.580	0.047	0.142
EX180°/s PT/BW (%)	Pretest	43.63 ± 12.08	50.85 ± 21.19	1.006 (0.400~1.603)	68.520 (0.000)	0.071 (0.792)	12.155 (0.001)
	Posttest	32.50 ± 8.38	23.50 ± 11.35				
	Mean difference	−11.13 ± 10.71	−27.34 ± 20.09				
	*t*(*p*)	5.091 (0.000)	6.666 (0.000)				
	Effect size (*η*^2^*_p_*)				0.598	0.002	0.209

Data are mean (standard deviation). PPRT, pedaling-based progressive resistance training; EX, extension; PT, peak torque; and BW, body weight. The effect sizes (Cohen’s *d*) were calculated comparing the pre- and posttest muscle strength means of both groups. *F*, repeated measures ANOVA. Significant: *p* < 0.05.

**Table 5:** The comparison of the hamstring muscle strength (*N* = 48).

Variable		PPRT Group (*n* = 24)	Control Group (*n* = 24)	Cohen’s *d* (95% CI)	Time	Group	Time × Group
					*F*(*p*)	*F*(*p*)	*F*(*p*)
FLX60°/s PT(N-M)	Pretest	25.33 ± 8.15	26.46 ± 11.62	0.743 (0.154~1.325)	6.686 (0.013)	2.160 (0.148)	6.660 (0.013)
	Posttest	25.32 ± 11.90	17.73 ± 5.05				
	Mean difference	0.03 ± 12.54	−8.73 ± 10.98				
	*t*(*p*)	0.003 (0.997)	3.893 (0.001)				
	Effect size (*η*^2^*_p_*)				0.127	0.045	0.126
FLX60°/s PT/BW (%)	Pretest	39.19 ± 9.66	46.16 ± 20.69	0.815 (0.154~1.325)	20.592 (0.000)	0.026 (0.872)	9.025 (0.004)
	Posttest	35.89 ± 10.90	29.93 ± 7.78				
	Mean difference	−3.30 ± 11.32	−15.69 ± 18.27				
	*t*(*p*)	1.428 (0.167)	4.470 (0.000)				
	Effect size(*η*^2^*_p_*)				0.309	0.001	0.164
FLX180°/s PT(N-M)	Pretest	19.71 ± 7.80	18.09 ± 9.52	0.003 (−0.563~0.569)	26.464 (0.000)	1.215 (0.276)	0.056 (0.814)
	Posttest	13.64 ± 5.64	11.44 ± 5.79				
	Mean difference	−6.06 ± 6.29	−6.09 ± 10.70				
	*t*(*p*)	4.725 (0.000)	3.148 (0.005)				
	Effect size(*η*^2^*_p_*)				0.365	0.026	0.001
FLX180°/s PT/BW (%)	Pretest	30.42 ± 10.09	32.01 ± 17.59	0.263 (−0.307~0.830)	30.902 (0.000)	0.014 (0.906)	0.890 (0.350)
	Posttest	21.00 ± 7.85	18.75 ± 9.96				
	Mean difference	−9.60 ± 8.57	−13.26 ± 17.78				
	*t*(*p*)	5.052 (0.000)	3.655 (0.001)				
	Effect size(*η*^2^*_p_*)				0.402	0.000	0.019

Data are mean (standard deviation). PPRT, the pedaling-based progressive resistance training; FLX, flexion; PT, peak torque; and BW, body weight. The effect sizes (Cohen’s *d*) were calculated comparing the pre- and posttest muscle strength means of both groups. *F*, repeated measures ANOVA. Significant: *p* < 0.05.

**Table 6:** The comparison of physical function (*N* = 48).

Variable		PPRT Group (*n* = 24)	Control Group (*n* = 24)	Cohen’s *d* (95% CI)	Time	Group	Time × Group
					*F*(*p*)	*F*(*p*)	*F*(*p*)
WOMAC Pain	Pretest	13.41 ± 2.74	13.50 ± 2.41	0.807 (0.214~1.392)	234.173 (0.000)	6.642 (0.013)	7.813 (0.008)
	Posttest	5.45 ± 0.88	8.00 ± 2.75				
	Mean difference	7.95 ± 2.42	5.50 ± 3.56				
	*t*(*p*)	16.095 (0.000)	7.562 (0.000)				
	Effect size (*η*^2^*_p_*)				0.836	0.126	0.145
WOMAC Stiffness	Pretest	5.41 ± 1.41	4.66 ± 1.49	0.423 (−0.152~0.993)	114.656 (0.000)	2.495 (0.121)	2.247 (0.141)
	Posttest	3.04 ± 0.99	2.87 ± 0.79				
	Mean difference	2.29 ± 1.04	1.75 ± 1.48				
	*t*(*p*)	10.617 (0.000)	5.627 (0.000)				
	Effect size (*η*^2^*_p_*)				0.714	0.051	0.047
WOMAC Physical function	Pretest	49.62 ± 7.02	47.33 ± 5.96	0.660 (0.075~1.239)	366.176 (0.000)	0.029 (0.866)	5.062 (0.029)
	Posttest	30.41 ± 6.88	32.16 ± 5.41				
	Mean difference	18.87 ± 5.62	14.58 ± 7.27				
	*t*(*p*)	17.541 (0.000)	10.651 (0.000)				
	Effect size (*η*^2^*_p_*)				0.888	0.001	0.099
WOMAC Total	Pretest	68.46 ± 9.002	65.50 ± 6.29	0.967 (0.363~1.561)	604.805 (0.000)	0.108 (0.744)	11.222 (0.002)
	Posttest	38.92 ± 6.846	43.04 ± 6.097				
	Mean difference	29.54 ± 6.6	22.46 ± 7.984				
	*t*(*p*)	21.927 (0.000)	13.781 (0.000)				
	Effect size (*η*^2^*_p_*)				0.929	0.002	0.196
TUG (s)	Pretest	12.96 ± 1.79	12.75 ± 2.12	0.596 (0.014~1.171)	106.67 (0.000)	0.946 (0.336)	4.216 (0.046)
	Posttest	9.32 ± 1.29	10.31 ± 1.57				
	Mean difference	3.64 ± 1.41	2.42 ± 2.51				
	*t*(*p*)	12.629 (0.000)	4.747 (0.000)				
	Effect size (*η*^2^*_p_*)				0.699	0.020	0.084

Data are mean (standard deviation). PPRT, pedaling-based progressive resistance training; WOMAC, the Western Ontario and McMaster Universities osteoarthritis index. The effect sizes (Cohen’s *d*) were calculated comparing the pre- and posttest muscle strength means of both groups. *F*, repeated measures ANOVA. Significant: *p* < 0.05.

## Data Availability

The original contributions presented in this study are included in the article. Further inquiries can be directed to the corresponding author.
